# Nucleus pulposus primary cilia alter their length in response to changes in extracellular osmolarity but do not control TonEBP-mediated osmoregulation

**DOI:** 10.1038/s41598-019-51939-7

**Published:** 2019-10-29

**Authors:** Hyowon Choi, Vedavathi Madhu, Irving M. Shapiro, Makarand V. Risbud

**Affiliations:** 1 Department of Orthopaedic Surgery, Sidney Kimmel Medical College, Thomas Jefferson University, Philadelphia, PA USA; 2 Graduate Program in Cell Biology and Regenerative Medicine, Thomas Jefferson University, Philadelphia, PA USA

**Keywords:** Cell signalling, Organelles

## Abstract

The nucleus pulposus (NP) cells adapt to their physiologically hyperosmotic microenvironment through Tonicity-responsive enhancer binding protein (TonEBP/nuclear factor of activated T-cell5 [NFAT5])-mediated osmoregulation. Primary cilia in different organs serve diverse roles including osmosensing, but its contribution to NP cell osmoadaptive response is unknown. A high percentage of cultured primary NP cells possessed primary cilia that changed length in response to osmotic stimuli. Stable silencing of *Intraflagellar Transport 88 (Ift88)* or *Kinesin Family Member 3* *A (Kif3a)* to inhibit the formation of primary cilia did not affect hyperosmotic upregulation of TonEBP. While Sh*Kif3a* blocked hyperosmotic increase of TonEBP-Transactivation Domain (TAD) activity, overall the knockdown of either gene did not alter the hyperosmotic status of proximal promoter activities and transcription of key TonEBP targets. On the other hand, a small decrease in TonEBP level under hypoosmotic condition was attenuated by *Ift88* or *Kif3a* knockdown. Noteworthy, none of the TonEBP target genes were responsive to hypoosmotic stimulus in control and *Ift88* or *Kif3a* knockdown cells, suggesting the primary role of TonEBP in the hyperosmotic adaptation of NP cells. Similarly, in *Kif3a* null mouse embryonic fibroblasts (MEFs), the overall TonEBP-dependent hyperosmotic responses were preserved. Unlike NP cells, TonEBP targets were responsive to hypoosmolarity in wild-type MEFs, and these responses remained intact in *Kif3a* null MEFs. Together, these results suggest that primary cilia are dispensable for TonEBP-dependent osmoadaptive response.

## Introduction

The nucleus pulposus (NP) is a gelatinous center-most structure of the intervertebral disc. It is surrounded by concentric layers of annulus fibrosus and sandwiched between cartilaginous endplates. The extracellular matrix of NP consists of collagens and abundant proteoglycans that are bound by sulfated glycosaminoglycan chains^1^. The high negative charge density of glycosaminoglycan molecules draws water and cations, in particular, Na^+^, into the NP tissue. This contributes to the elevated osmotic swelling pressure within the tissue compartment and provides the tissue with its ability to resist daily spinal loadings^1,2^. The NP matrix is, therefore, hyperosmotic with estimated tissue osmolarity raging from 430 to 496 mOsm/kg H_2_O^3,4^. With aging and degeneration of the tissue, the aberrant changes in cell phenotype lead to altered extracellular matrix composition, decreased osmotic pressurization of the NP, and compromised the mechanical function of the tissue^1,5,6^. Therefore, adaptation to their osmodynamic niche for survival and function is crucial for the resident NP cells.

Tonicity-responsive enhancer binding protein (TonEBP/nuclear factor of activated T-cell5 [NFAT5]) is a Rel homology transcription factor that has been well characterized for its osmolarity-dependent function in mammalian cells including NP cells^7–14^. In response to a hyperosmotic stimulus, TonEBP transcriptionally controls the expression of several osmoprotective genes including taurine transporter (TauT), betaine-GABA transporter (BGT-1), sodium/myo-inositol co-transporter (SMIT), and aldose reductase (AR), all of which are essential regulators of intracellular levels of non-ionic osmolytes^15–18^. Additionally, TonEBP transcriptionally increases the expression of heat shock protein-70 (HSP-70) to maintain proper protein folding, cellular trafficking, and degradation of misfolded proteins under hyperosmotic conditions^19,20^. It was recently shown that TonEBP is highly expressed in NP as well as notochord cells of developing mouse embryo, supporting its importance in NP development and maintenance^21^.

Primary cilia found in most types of mammalian cells have diverse roles, including modulation of key signaling pathways^22–28^. They can also function as a sensory organelle that relays extracellular stimuli, such as changes in osmolarity, to intracellular signaling pathways^29–38^. In *Caenorhabditis elegans*, OSM-9, a homolog of mammalian transient receptor potential channel vanilloid subfamily (TRPV), and OCR-2, OSM-9/capsaicin receptor related TRPV channel, that are localized in primary cilia are important for sensing extracellular hyperosmotic stimulus^35^. Similarly, primary cilia of mammalian cholangiocytes and articular chondrocytes are shown to link extracellular hypoosmotic stimulus to intracellular calcium signaling pathway^36,37^. In a recent report, Siroky *et al*. showed that renal epithelial cells without primary cilia have weakened induction of TonEBP target genes *AR/Akr1b3* as well as *Bgt1* under hyperosmotic conditions^38^. Although the role of TonEBP in modulating osmoresponse in NP cells has been well studied, it is unknown whether primary cilia contribute to this process. The objective of this study was to investigate if primary cilia function as osmosensory organelles in NP cells. Specifically, we examined if primary cilia control TonEBP-mediated osmoadaptive response through loss-of-function studies measuring the expression of TonEBP and its target genes after inhibition of primary cilia formation. Furthermore, we confirmed our findings in NP cells using *Kif3a* null mouse embryonic fibroblasts (MEFs) that are completely devoid of primary cilia.

## Results

### The length of primary cilia in NP cells is responsive to changes in extracellular osmolarity

**Figure 1:**
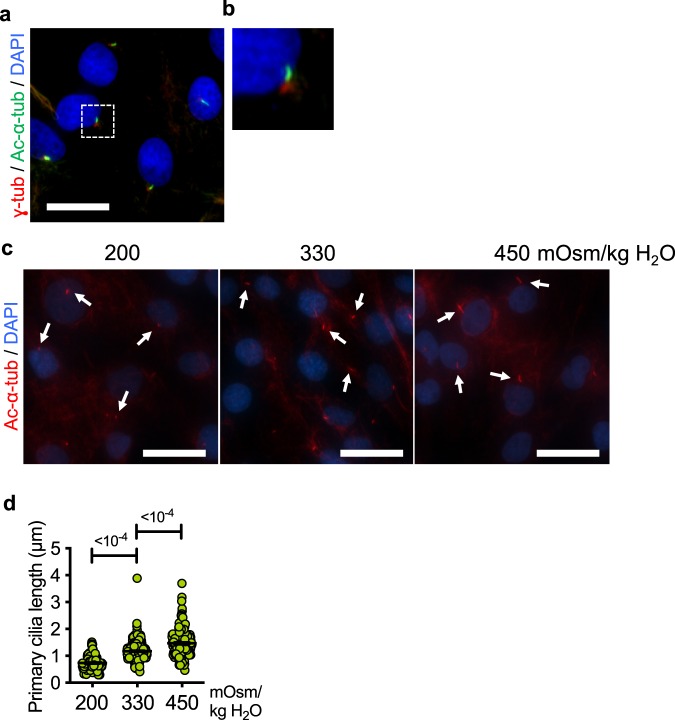
NP cell primary cilia modulate their lengths in response to changes in extracellular osmolarity. (**a**) Immunofluorescence staining of acetylated α-tubulin (green) and γ-tubulin (red) to mark primary cilia axoneme and basal bodies, respectively, in primary rat NP cells. Scale bar = 30 μm. (**b**) Zoomed-in image of a primary cilium from the area demarcated by the white square in panel (**a**). (**c**,**d**) Primary cilia of rat NP cells cultured under different osmotic conditions for 24 h were visualized by immunofluorescence staining of acetylated α-tubulin. (**c**) The lengths of primary cilia increase in response to increased osmolarity (450 mOsm/kg H_2_O) compared to isoosmotic control (330 mOsm/kg H_2_O) conditions, whereas they appear shorter under hypoosmotic conditions (200 mOsm/kg H_2_O). White arrows mark primary cilia. Scale bar = 50 μm. (**d**) Quantification of primary cilium length was done using ImageJ software. (n = 3 experiments; at least 150 cells/group) Data are represented as scatter plots (mean ± SEM). One-way ANOVA with Dunnett’s multiple comparison test was used to determine statistical significance.

Primary cilia were visualized in cultured primary rat NP cells by co-immunostaining acetylated α-tubulin and γ-tubulin, labeling ciliary axoneme and basal bodies, respectively (Fig. 1a,b). Previous studies showed that the length of primary cilia in different types of cells changed in response to extracellular stimuli^39–41^. To examine if primary cilia in NP cells respond to extracellular osmotic stimulus, we cultured NP cells under different osmotic conditions and measured the length of the cilia. The average length of primary cilia was significantly shorter under hypoosmotic condition (200 mOsm/kg H_2_O) compared to isoosmotic (330 mOsm/kg H_2_O) condition (Fig. 1c,d; *p* < 0.0001 for both 200 mOsm/kg H_2_O and 450 mOsm/kg H_2_O groups). On the other hand, the length of primary cilia increased under hyperosmotic condition (450 mOsm/kg H_2_O), suggesting that the primary cilia in NP cells are sensitive to changes in extracellular osmolarity.

### Inhibition of primary cilia formation in NP cells does not affect TonEBP expression in hyperosmotic condition

**Figure 2:**
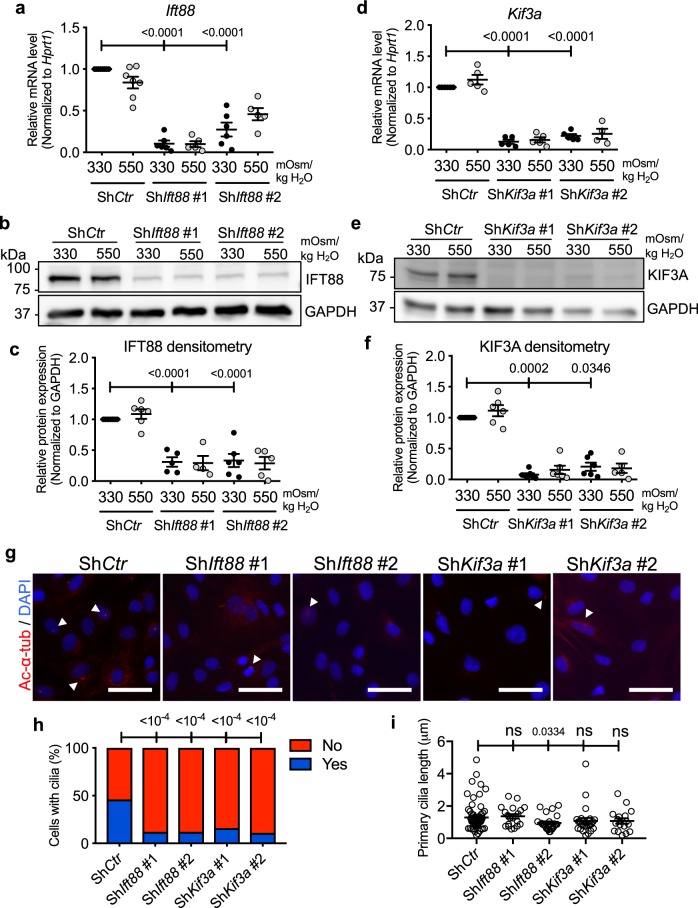
Stable knockdown of *Ift88* or *Kif3a* inhibits formation of NP cell primary cilia. (**a**) *Ift88* mRNA levels in NP cells transduced with control (Sh*Ctr*) or two different Sh*Ift88* clones were measured by qRT-PCR to confirm the knockdown (n ≥ 5). (**b**) Western blot image showing significant reduction of IFT88 protein levels after the knockdown of *Ift88*. (**c**) Densitometry analyses of Western blots confirm significant knockdown of IFT88 (n ≥ 5). (**d**–**f**) qRT-PCR, Western blot, and corresponding densitometry analyses, show significant downregulation of KIF3A after stable knockdown using two different Sh*Kif3a* clones (n ≥ 4). (**g**) Acetylated α-tubulin immunofluorescence staining after lentiviral transduction of Sh*Ift88* or Sh*Kif3a* shows inhibition of primary cilia formation in majority of rat NP cells. Scale bar = 75 μm. White arrowheads point to primary cilia. (**h**,**i**) Quantitation of percentage of NP cells with primary cilia and primary cilium length after stable silencing of *Ift88* or *Kif3a* (n = 3; at least 150 cells/group). Data are represented as scatter plots (mean ± SEM). ns = not significant. One-way ANOVA or Kruskal-Wallis test with Sidak’s, Holm-Sidak’s, or Dunn’s multiple comparison test was used based on the distribution of the data to determine statistical significance. For statistical comparison of the percentages of NP cells with primary cilia, Fisher’s exact test was used. Western blot images were cropped and acquired under same experimental conditions. See Supplementary Fig. S1–1 for un-cropped Western blot images.

The function of primary cilia as an osmosensor has been observed in *C. elegans* as well as in some types of mammalian cells, including renal tubular epithelial cells, articular chondrocytes, and cholangiocytes^35–38^. NP cells reside in an osmotically active microenvironment due to high proteoglycan content of the NP matrix and dynamic loading of the spine. We examined if primary cilia of the NP cells play a role in sensing extracellular osmolarity and mediating cellular osmotic response. We inhibited formation of primary cilia in NP cells by performing stable knockdown of *Ift88*, a component of complex B of the intraflagellar transport (IFT) particles^42–45^ (Fig. 2a–c; Supp. Fig. S1–1), or *Kif3a*, a motor subunit of kinesin 2^46^ (Fig. 2d–f), both of which are necessary for ciliogenesis. Lentiviral transduction of NP cells with shRNA against *Ift88* or *Kif3a* resulted in a significant decrease in the transcript and protein levels of IFT88 (Fig. 2a–c; *p* < 0.0001 for all statistical comparisons) or KIF3A (Fig. 2d–f; *p* < 0.0001 for Sh*Kif3a* #1 and #2 isoosmotic groups in Fig. 2d; *p* = 0.0002 for Sh*Kif3a* #1 isoosmotic group, *p* = 0.346 for Sh*Kif3a* #2 isoosmotic group in Fig. 2f; Supp. Fig. S1–1), respectively. Stable silencing of either gene resulted in a decreased number of cells with primary cilia (Fig. 2g). Quantification of the number of cells with primary cilia confirmed this result (Fig. 2h; *p* < 0.0001 for all groups). Overall, the lengths of primary cilia that remained after the stable silencing of *Ift88* or *Kif3a* were not significantly different from that of the control cells (Fig. 2i; *p* = 0.0334 for Sh*Ift88* #2, all other groups were statistically not significant).

**Figure 3:**
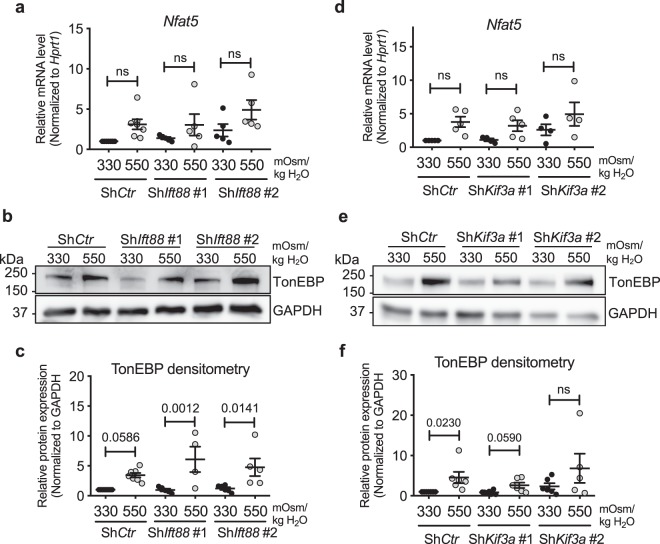
Knockdown of *Ift88* or *Kif3a* in NP cells does not affect hyperosmotic upregulation of TonEBP. (**a**) *TonEBP/Nfat5* mRNA levels in NP cells with *Ift88* knockdown (n ≥ 5). (**b**) Western blot image showing increased TonEBP expression in response to hyperosmolarity (550 mOsm/kg H_2_O) independently of *Ift88* knockdown. (**c**) Densitometry analyses of TonEBP with *Ift88* knockdown (n ≥ 4). (**d**) *TonEBP/Nfat5* mRNA levels in NP cells with *Kif3a* knockdown (n ≥ 3). (**e**) Western blot image showing that hyperosmotic induction of TonEBP is maintained after *Kif3a* knockdown. (**f**) Densitometry analyses of TonEBP after *Kif3a* knockdown (n ≥ 4). Data are represented as scatter plots (mean ± SEM). ns = not significant. One-way ANOVA or Kruskal-Wallis test with Sidak’s or Dunn’s multiple comparison test was used based on the distribution of the data to determine statistical significance. Western blot images were cropped and acquired under same experimental conditions. See Supplementary Fig. S1-1 for un-cropped Western blot images.

To determine if inhibition of primary cilia formation resulted in dysregulation of cellular osmoresponse, we stably silenced *Ift88* or *Kif3a* and measured the expression of TonEBP/NFAT5, a transcription factor crucial for osmoregulation in NP cells^8–11^. While there was a trend of increase in *TonEBP/Nfat5* transcript levels under hyperosmotic conditions (550 mOsm/kg H_2_O), this increase did not reach a statistical significance in both control and *Ift88* knockdown cells (Fig. 3a; all analyses were statistically not significant). Hyperosmotic increase in TonEBP protein levels was unaffected by *Ift88* knockdown (Fig. 3b,c; *p* = 0.0586 for Sh*Ctr* 550 mOsm/kg H_2_O, *p* = 0.0012 for Sh*Ift88* #1 550 mOsm/kg H_2_O, *p* = 0.0141 for Sh*Ift88* #2 550 mOsm/kg H_2_O; Supp. Fig. S1–1). Similarly, when *Kif3a* was stably silenced, the trend of increase in *TonEBP/Nfat5* transcript levels and the upregulation of TonEBP protein under hyperosmotic condition were maintained (Fig. 3d–f; Statistical non-significance for all analyses in Fig. 3d; *p* = 0.023 for Sh*Ctr* 550 mOsm/kg H_2_O, *p* = 0.059 for Sh*Kif3a* #1 550 mOsm/kg H_2_O; Supp. Fig. S1–1). Taken together, our data suggest that inhibition of primary cilia formation in NP cells does not affect their TonEBP expression.

### Primary cilia do not modulate TonEBP activity and TonEBP target gene expression in NP cells under hyperosmotic conditions

**Figure 4:**
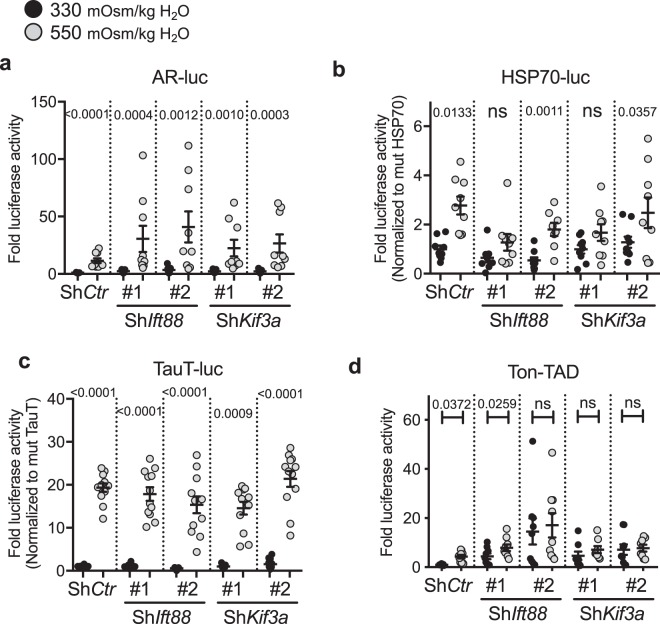
Sh*Ift88* or Sh*Kif3a* does not affect the promoter activities of TonEBP target genes under hyperosmotic conditions. (**a**–**c**) Proximal promoter activities of *AR* (**a**), *HSP-70* (**b**), and *TauT* (**c**) in response to hyperosmotic stimuli (550 mOsm/kg H_2_O) with or without Sh*Ift88* or Sh*Kif3a*. The promoter activities of TonEBP target genes are mostly unaffected by Sh*Ift88* or Sh*Kif3a*. (**d**) Sh*Kif3a* as well as one of the Sh*Ift88* clones prevent further upregulation of TonEBP-TAD (Ton-TAD) activity in response to hyperosmolarity. Interestingly, the levels of TonEBP-TAD activity under isoosmotic conditions are increased with *ShIft88* or *ShKif3a*. Data are represented as scatter plots (mean ± SEM) (n ≥ 3 with 3 technical replicates per biological replicate). ns = not significant. One-way ANOVA or Kruskal-Wallis test with Sidak’s or Dunn’s multiple comparison test was used based on the distribution of the data to determine statistical significance.

Hyperosmotic stimulus leads to the increased transcriptional activity of TonEBP in the nucleus^9,11,47^. We investigated if primary cilia controlled TonEBP activity without affecting its expression. Changes in proximal promoter activities of well-characterized TonEBP target genes including, *AR* (aldose reductase), *HSP-70* and *TauT* (taurine transporter) were measured in NP cells transfected with shRNA against *Ift88* or *Kif3a* (Fig. 4a–c). These promoters contain highly conserved TonEBP-binding sites that are active in NP cells^10,15,19,20,47,48^. Hyperosmotic (550 mOsm/kg H_2_O) increase in the *AR* (Fig. 4a; *p* < 0.0001) and *TauT* (Fig. 4c; *p* < 0.0001) promoter activities was not affected by either Sh*Ift88* (*p* = 0.0004 for clone #1, *p* = 0.0012 for clone #2 for AR-luc; *p* < 0.0001 for both clones for TauT-luc) or Sh*Kif3a* (*p* = 0.001 for clone #1, *p* = 0.0003 for clone #2 for AR-luc; *p* = 0.0009 for clone #1 and *p* < 0.0001 for clone #2 for TauT-luc). Similarly, the *HSP-70* promoter activity increased under hyperosmotic conditions with or without Sh*Ift88* or Sh*Kif3a*. While one of the Sh*Ift88* and Sh*Kif3a* clone groups did not reach a statistical significance, the trend of hyperosmotic induction of *HSP-70* promoter activity was maintained (Fig. 4b; *p* = 0.0011 for Sh*Ift88* #2, *p* = 0.0357 for Sh*Kif3a* #2). In addition, we measured the activity of the TonEBP transactivation domain (TAD) in response to the hyperosmotic stimulus (550 mOsm/kg H_2_O) with or without Sh*Ift88* and Sh*Kif3a*. While Sh*Ift88* #2 inhibited the hyperosmotic increase of TonEBP-TAD activity, the other clone had no effect (Fig. 4d; *p* = 0.0259 for Sh*Ift88* #1). Both clones of Sh*Kif3a* prevented a further increase of TonEBP-TAD activity in response to the hyperosmotic stimulus. However, the average level of TonEBP-TAD activity under hyperosmotic conditions was similar or higher in knockdown cells when compared to Sh*Ctr* group, suggesting that the overall TonEBP-TAD activity was unaffected by Sh*Ift88* or Sh*Kif3a* (Fig. 4d).

**Figure 5:**
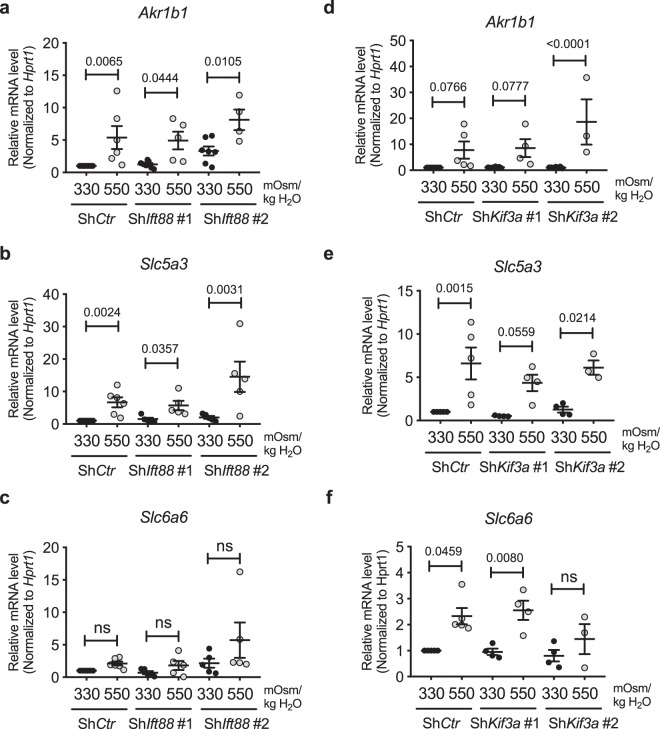
Hyperosmotic upregulation of TonEBP target gene expression is not affected by inhibition of primary cilia formation in NP cells. (**a**–**c**) mRNA levels of TonEBP targets, AR*/Akr1b1*, *SMIT/Slc5a3* and *TauT/Slc6a6*, showing that stable silencing of *Ift88* does not affect their response to hyperosmotic stimuli (550 mOsm/kg H_2_O) (n ≥ 5). (**d**–**f**) mRNA levels of *AR/Akr1b1*, *SMIT/Slc5a3* and *TauT/Slc6a6* demonstrate that *Kif3a* knockdown also does not influence hyperosmotic upregulation of these genes (n ≥ 3). Data are represented as scatter plots (mean ± SEM). ns = not significant. One-way ANOVA or Kruskal-Wallis test with Sidak’s or Dunn’s multiple comparison test was used based on the distribution of the data to determine statistical significance.

To confirm that TonEBP-mediated hyperosmotic response was independent of primary cilia, we measured mRNA levels of TonEBP targets in NP cells with *Ift88* or *Kif3a* knockdown. *AR/Akr1b1*, *TauT/Slc6a6* as well as sodium/myo-inositol co-transporter (*SMIT/Slc5a3*) are osmolarity-dependent target genes of TonEBP^15–18^. *Ift88* knockdown did not affect the hyperosmotic (550 mOsm/kg H_2_O) increase in the expression of *AR/Akr1b1* and *SMIT/Slc5a3* genes (Fig. 5a,b; *p* = 0.0065 for Sh*Ctr*, *p* = 0.0444 for Sh*Ift88* #1, *p* = 0.0105 for Sh*Ift88* #2 in Fig. 5a; *p* = 0.0024 for Sh*Ctr*, *p* = 0.0357 for Sh*Ift88* #1, *p* = 0.0031 for Sh*Ift88* #2 in Fig. 5b). While the increase in *TauT/Slc6a6* levels in response to hyperosmotic stimulus did not reach a statistical significance, the trend of increase was unaffected by *Ift88* knockdown (Fig. 5c). Likewise, stable silencing of *Kif3a* did not affect the induction of *SMIT*/*Slc5a3* and *AR/Akr1b1* in response to hyperosmolarity (550 mOsm/kg H_2_O) (Fig. 5d, e; *p* = 0.0766 for Sh*Ctr*, *p* = 0.0777 for Sh*Kif3a* #1, *p* < 0.0001 for Sh*Kif3a* #2 in Fig. 5d; *p* = 0.0015 for Sh*Ctr*, *p* = 0.0559 for Sh*Kif3a* #1, *p* = 0.0214 for Sh*Kif3a* #2 in Fig. 5e). In addition, the hyperosmotic increase of *TauT/Slc6a6* expression was preserved with Sh*Kif3a*, except one of the clones (Fig. 5f; *p* = 0.0459 for Sh*Ctr*, *p* = 0.008 for Sh*Kif3a* #1). Taken together, the gene expression data showed that inhibition of primary cilia formation in NP cells did not affect the overall TonEBP transcriptional activity or the target gene expression under hyperosmotic conditions.

### The absence of primary cilia has a minor effect on hypoosmotic downregulation of TonEBP and its targets in NP cells

**Figure 6:**
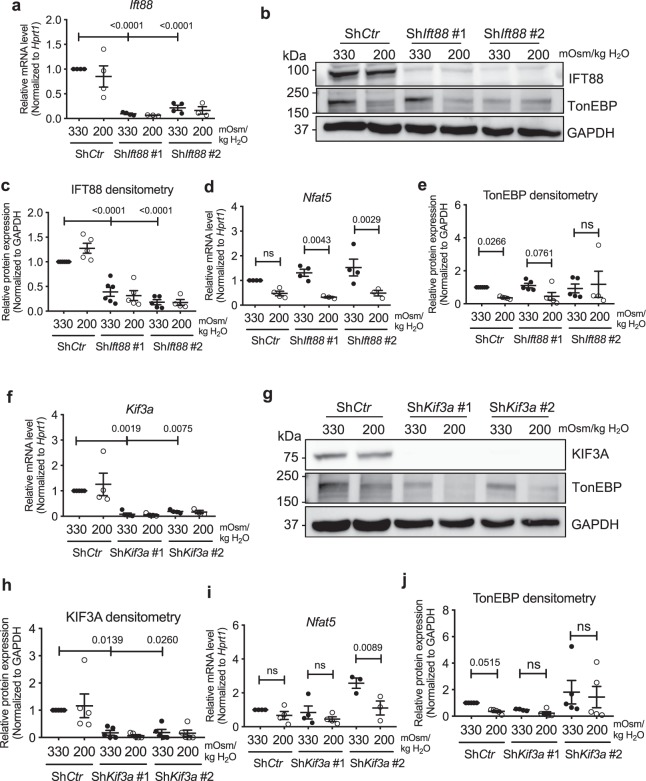
Inhibition of primary cilia formation in NP cells have minimal effect on hypoosmotic downregulation of TonEBP levels. (**a**–**c**) qRT-PCR, Western blot and densitometry analyses demonstrating IFT88 levels under hypoosmotic conditions (200 mOsm/kg H_2_O) after stable knockdown of *Ift88*. (**d**) *TonEBP/Nfat5* expression in NP cells with *Ift88* knockdown under hypoosmotic conditions (n ≥ 3). (**d**) Hypoosmotic decrease in TonEBP protein levels is attenuated by *Ift88* knockdown (n ≥ 4). (**f**,**g**) KIF3A mRNA and protein levels in NP cells with *Kif3a* knockdown. (**h**) *TonEBP/Nfat5* mRNA expression in NP cells with *Kif3a* knockdown under hypoosmotic conditions (n ≥ 3). (**i**) Hypoosmotic downregulation of TonEBP is blunted after *Kif3a* knockdown (n ≥ 4). Data are represented as scatter plots (mean ± SEM). ns = not significant. One-way ANOVA or Kruskal-Wallis test with Sidak’s or Dunn’s multiple comparison test was used based on the distribution of the data to determine statistical significance. See Supplementary Figs S1 and 2 for un-cropped Western blot images.

The tissue osmotic conditions of the NP compartment undergo diurnal changes due to dynamic loading and unloading of the spine. During disc degeneration, the breakdown of the proteoglycan matrix by proteases decreases the tissue osmolarity^49–51^. Consequently, NP cells need to maintain their function and integrity under both hyper- and hypo-osmotic conditions^52,53^. We, therefore, assessed the effects of inhibiting primary cilia formation in NP cells on TonEBP expression under hypoosmotic conditions (200 mOsm/kg H_2_O). NP cells with *Ift88* (Fig. 6a–c; *p* < 0.0001 for all analyses in Fig. 6a,c; Supp. Figs S1 and 2) or *Kif3a* (Fig. 6f–h; *p* = 0.0019 for Sh*Kif3a* #1, *p* = 0.0075 for Sh*Kif3a* #2 in Fig. 6f; *p* = 0.0139 for Sh*Kif3a* #1, p = 0.026 for Sh*Kif3a* #2 in Fig. 6h) knockdown showed significant hypoosmotic decrease in *TonEBP/Nfat5* transcript, compared to control cells that showed similar decreasing trend (Fig. 6d,i; *p* = 0.0043 for Sh*Ift88* #1, *p* = 0.0029 for Sh*Ift88* #2 in Fig. 6d; *p* = 0.0089 for Sh*Kif3a* #2 in Fig. 6i). On the other hand, a small but significant decrease in TonEBP protein levels seen in control cells under the hypoosmotic condition was attenuated following knockdown of *Ift88* (Fig. 6b,e; *p* = 0.0266 for Sh*Ctr*, *p* = 0.0761 for Sh*Ift88* #1) or *Kif3a* (Fig. 6g,j; *p* = 0.0515 for Sh*Ctr*; Supp. Figs S1 and 2). Although the changes in mRNA and protein levels in response to hypoosmotic stimuli were somewhat opposing in cells lacking primary cilia, these results suggested that primary cilia may play a minor role in maintaining TonEBP levels in NP cells under hypoosmotic conditions.

**Figure 7:**
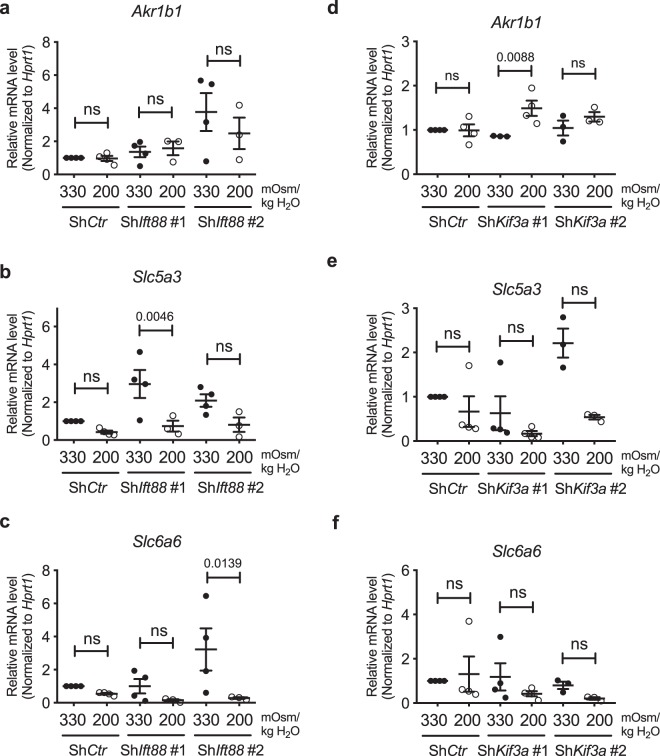
TonEBP target gene expression under hypoosmotic conditions is unaffected by inhibition of primary cilia formation in NP cells. (**a**–**f**) mRNA levels of *AR/Akr1b1, SMIT/Slc5a3* and *TauT/Slc6a6* under hypoosmotic conditions (200 mOsm/kg H_2_O) following knockdown of *Ift88* (**a**–**c**) or *Kif3a* (**d**–**f**). Stable silencing of either *Ift88* or *Kif3a* does not affect the trend of downregulation in *SMIT/Slc5a3* and *TauT/Slc6a6* under hypoosmotic conditions (n ≥ 3). Data are represented as scatter plots (mean ± SEM). ns = not significant. One-way ANOVA or Kruskal-Wallis test with Sidak’s or Dunn’s multiple comparison test was used based on the distribution of the data to determine statistical significance.

We then measured mRNA levels of TonEBP targets to investigate if the small changes in TonEBP levels in cells without primary cilia had functional implications in overall hypoosmotic cellular response. *AR/Ark1b1, SMIT/Slc5a3*, and *TauT/Slc6a6* were not affected by stable knockdown of *Ift88* or *Kif3a* (Fig. 7a–f; all statistical analyses were not significant in Fig. 7a,e,f; *p* = 0.0046 for Sh*Ift88* #1 in Fig. 7b; non-significant for Sh*Ctr* and Sh*Ift88* #1, *p* = 0.0139 for Sh*Ift88* #2 in Fig. 7c; non-significant for Sh*Ctr* and Sh*Kif3a* #2, *p* = 0.0088 for Sh*Kif3a* #1 in Fig. 7d). Interestingly, none of these osmotic targets showed a significant decrease in their expression under hypoosmotic conditions, and this trend was not affected by either *Ift88* or *Kif3a* knockdown (Fig. 7a–f). Taken together, our results showed that primary cilia may have a small role in modulating hypoosmotic levels of TonEBP, but this did not lead to any appreciable effect on target gene expression.

### TonEBP expression and activity are not mediated by primary cilia in mouse embryonic fibroblasts

**Figure 8:**
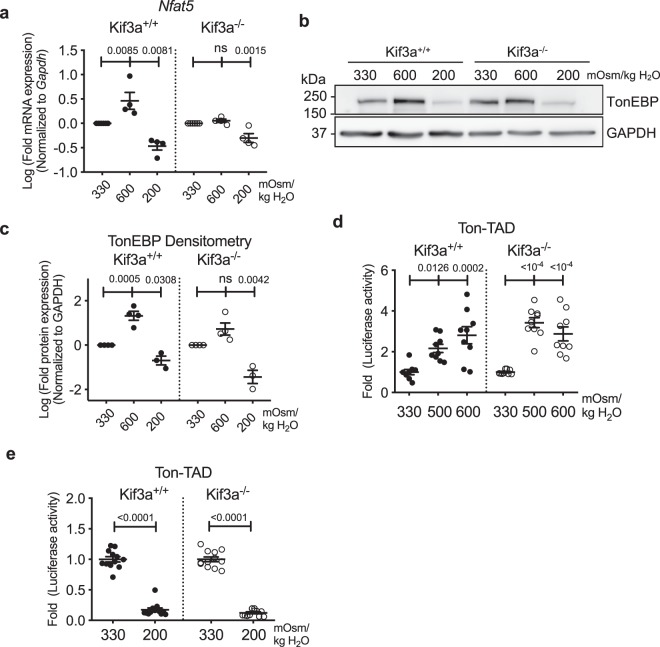
The absence of primary cilia in *Kif3a* null MEFs has a small effect on hyperosmotic induction of TonEBP without influencing its TAD activity (**a**) *TonEBP/Nfat5* gene expression in wild-type and *Kif3a* null (*Kif3a*^−/−^) MEFs under hyper- (600 mOsm/kg H_2_O) and hypoosmotic (200 mOsm/kg H_2_O) conditions. (**b**,**c**) Western blot image and corresponding densitometry analyses showing TonEBP levels under different osmotic conditions in wild-type and *Kif3a* null MEFs. *Kif3a* null MEFs show slightly attenuated hyperosmotic increase but unaffected hypoosmotic decrease in TonEBP expression. (**d**,**e**) TonEBP-TAD (Ton-TAD) activity in wild-type and *Kif3a* null MEFs under hyperosmotic (**d**) and hypoosmotic (**e**) conditions (n = 4). Similar to wild-type cells, *Kif3a* null MEFs show increase and decrease in TonEBP-TAD activity under hyperosmotic and hypoosmotic conditions, respectively (n ≥ 3 with 3 technical replicates per biological replicate). Data are represented as scatter plots (mean ± SEM). ns = not significant. One-way ANOVA or Kruskal-Wallis test with Sidak’s or Dunn’s multiple comparison test was used based on the distribution of the data to determine statistical significance. Western blot images were cropped and acquired under same experimental conditions. See Supplementary Figs S1 and 3 for un-cropped Western blot images.

Similar to NP cells, mouse embryonic fibroblasts (MEFs) induce TonEBP-dependent osmotic response under hyperosmotic conditions^8^. Parallel experiments were performed using *Kif3a* null MEFs that completely lack primary cilia to further confirm that primary cilia do not control TonEBP-dependent osmotic response^54^. As reported in other studies, TonEBP mRNA (Fig. 8a; *p* = 0.0085) and protein (Fig. 8b,c; *p* = 0.0005) levels were significantly upregulated in wild-type MEFs under hyperosmotic conditions (600 mOsm/kg H_2_O). On the other hand, the increase of TonEBP expression in *Kif3a* null MEFs under hyperosmotic conditions did not reach a statistical significance (Fig. 8a–c). In response to hypoosmotic conditions (200 mOsm/kg H_2_O), both wild-type and *Kif3a* null MEFs downregulated TonEBP transcript and protein levels (Fig. 8a–c; *p* = 0.0081 for wild-type MEFs, *p* = 0.0015 for *Kif3a* null MEFs in Fig. 8a; *p* = 0.0308 for wild-type MEFs, *p* = 0.0042 for *Kif3a* null MEFs in Fig. 8c; Supp. Figs S1 and 3).

To investigate if cilia controlled osmolarity-dependent TonEBP activity in MEFs, we measured the activation status of TonEBP-TAD in wild-type and *Kif3a* null MEFs under different osmotic conditions. Both wild-type and *Kif3a* null MEFs increased and decreased TonEBP-TAD activities under hyperosmotic conditions (600 mOsm/kg H_2_O) (Fig. 8d; *p* = 0.0126 for 500 mOsm/kg H_2_O, *p* = 0.0002 for 600 mOsm/kg H_2_O in wild-type MEFs; *p* < 0.0001 for both hyperosmotic conditions in *Kif3a* null MEFs), and hypoosmotic conditions (200 mOsm/kg H_2_O) (Fig. 8e; *p* < 0.0001 for both wild-type and *Kif3a* null MEFs), respectively. These results suggested that the effect of osmolarity on TonEBP-TAD activity remained unaffected in the absence of primary cilia.

**Figure 9:**
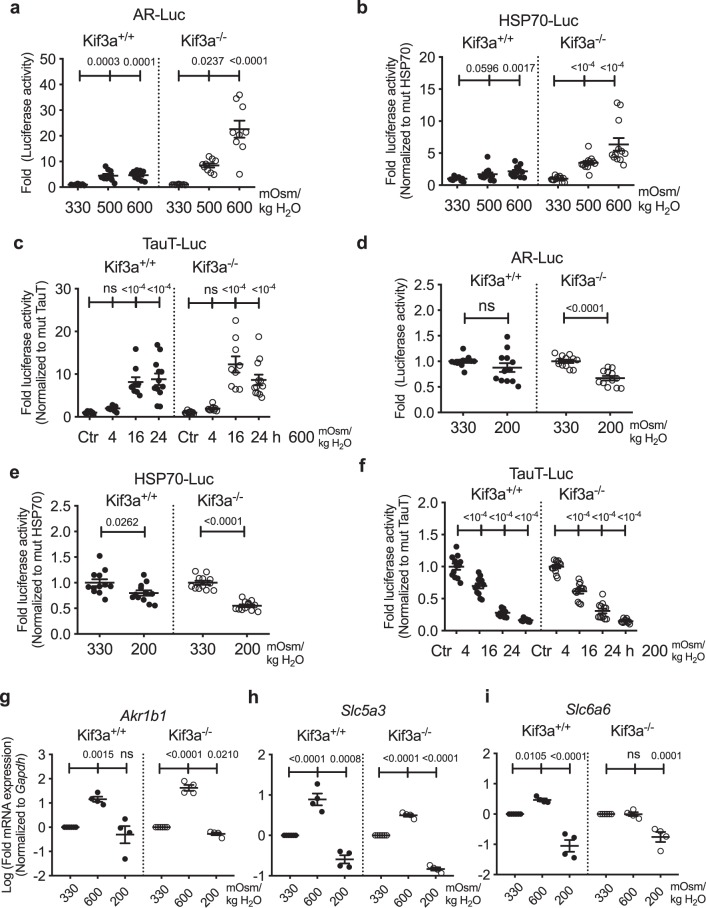
The absence of primary cilia does not affect TonEBP activity and target gene expression in MEFs (**a**–**c**) Proximal promoter activities of *AR*, *HSP-70* and *TauT* in wild-type and *Kif3a* null MEFs show that hyperosmotic induction (600 mOsm/kg H_2_O) of these promoter activities are intact in null MEFs. (**d**–**f**) *AR* promoter activity is unaffected in wild-type MEFs but decreased in *Kif3a* null MEFs under hypoosmotic conditions (200 mOsm/kg H_2_O). Hypoosmotic decrease in the promoter activities of *HSP-70 and TauT* are maintained in *Kif3a* null MEFs. (n ≥ 3 with 3 technical replicates per biological replicate). (**g**,**h**) The changes in expression of *AR/Akr1b1* (**g**) and *SMIT/Slc5a3* (**h**) under different osmotic conditions are similar between wild-type and *Kif3a* null MEFs (n = 4). (**i**) Hyperosmotic induction of *TauT/Slc6a6* gene expression observed in wild-type MEFs is absent in *Kif3a* null MEFs. *TauT/Slc6a6* gene expression was downregulated in both wild-type and *Kif3a* null MEFs under hypoosmotic conditions (n = 4). Data are represented as scatter plots (mean ± SEM). ns = not significant. One-way ANOVA or Kruskal-Wallis test with Sidak’s or Dunn’s multiple comparison test was used based on the distribution of the data to determine statistical significance.

We then measured the activities of TonEBP responsive reporters and the level of TonEBP target genes in wild-type and *Kif3a* null MEFs to determine if the presence of primary cilia affected TonEBP transcriptional activity. Hyperosmotic stimulus (600 mOsm/kg H_2_O) significantly increased *AR* and *HSP-70* promoter activities in both wild-type and *Kif3a* null MEFs (Fig. 9a,b; *p* = 0.0003 for 500 mOsm/kg H_2_O, *p* = 0.0001 for 600 mOsm/kg H_2_O for wild-type MEFs, *p* = 0.0237 for 500 mOsm/kg H_2_O, *p* < 0.0001 for 600 mOsm/kg H_2_O for *Kif3a* null MEFs in Fig. 9a; *p* = 0.0596 for 500 mOsm/kg H_2_O, *p* = 0.0017 for 600 mOsm/kg H_2_O for wild-type MEFs, *p* < 0.0001 for hyperosmolarity in *Kif3a* null MEFs in Fig. 9b). Likewise, the temporal response of *TauT* promoter to the hyperosmotic stimulus was similar in both wild-type and *Kif3a* null MEFs, where a robust activation at 16 h post-treatment was observed (Fig. 9c; *p* < 0.0001 for 16 h and 24 h in both wild-type and *Kif3a* null MEFs). Interestingly, the *AR* promoter activity was insensitive to the hypoosmotic stimulus (200 mOsm/kg H_2_O) in wild-type cells, while it was significantly downregulated in *Kif3a* null MEFs (Fig. 9d; p < 0.0001). Both wild-type and *Kif3a* null MEFs showed a hypoosmotic reduction in *HSP70* promoter activity (Fig. 9e; *p* = 0.0262 for wild-type, *p* < 0.0001 for *Kif3a* null MEFs). A gradual and time-dependent decrease in *TauT* promoter activity was also observed under hypoosmotic conditions (Fig. 9f; *p* < 0.0001).

A qRT-PCR analysis was performed to measure the expression of select TonEBP target genes in MEFs under different osmotic conditions. *AR/Akr1b1* was significantly upregulated in both wild type and *Kif3a* null MEFs under hyperosmotic conditions (600 mOsm/kg H_2_O) (Fig. 9g; *p* = 0.0015 for wild-type, *p* < 0.0001 for null MEFs). In agreement with its promoter activity, *AR/Akr1b1* expression was not changed in wild-type MEFs but was significantly decreased in *Kif3a* null MEFs in response to the hypoosmotic stimulus (200 mOsm/kg H_2_O) (Fig. 9g; *p* = 0.021). *SMIT/Slc5a3* expression in both wild-type and *Kif3a* null MEFs was significantly increased and decreased under hyperosmotic and hypoosmotic conditions, respectively (Fig. 9h; *p* < 0.0001 for hyperosmotic condition in wild-type and *Kif3a* null MEFs, *p* = 0.0008 for hypoosmotic condition in wild-type MEFs, *p* < 0.0001 for hypoosmotic condition in *Kif3a* null MEFs). Interestingly, unlike in wild-type MEFs, *TauT/Slc6a6* mRNA levels were not induced by hyperosmolarity in *Kif3a* null MEFs (Fig. 9i; *p* = 0.0105 in wild-type MEFs). The hypoosmotic decrease in *TauT/Slc6a6* levels was maintained in both wild-type and *Kif3a* null MEFs (Fig. 9i; *p* < 0.0001 in wild-type, *p* = 0.0001 in null MEFs). Taken together, the osmotic regulation of TonEBP activity in MEFs remained largely unchanged in the absence of primary cilia.

## Discussion

Primary cilia serve diverse physiological functions that are well-conserved from *C. elegans* to vertebrates. Their sensory function in various cell types and organ systems is essential in multiple biological processes such as development and maintenance of tissue homeostasis. There are only a few studies examining the presence of primary cilia in the disc tissues^55^. However, few studies have investigated the role of primary cilia in the intervertebral disc^56^. A few studies have suggested that primary cilia can function as osmosensors in mammalian cells^36–38^. In rat cholangiocytes or porcine articular chondrocytes, removal of primary cilia inhibits intracellular Ca^2+^ increase in response to hypoosmotic stimuli^36,37^. In addition, renal epithelial cells depleted of primary cilia cannot induce TonEBP target genes, *AR/Akr1b3*, and *Bgt1*, under hyperosmotic conditions^38^. These cell types are frequently exposed to dynamic changes in extracellular osmolarity, similar to those experienced by the NP cells. Importantly, since NP tissue is physiologically hyperosmotic, osmoadaptation is a key survival mechanism for the resident cells. We, therefore, investigated whether primary cilia in NP cells have an osmosensory function and if the activity of TonEBP, a key osmoregulatory transcription factor, is dependent on primary cilia. We show, for the first time, that NP cell primary cilia do not have the osmoregulatory function, although they alter their lengths in response to changes in extracellular osmolarity.

In many cellular pathways that are mediated by primary cilia, the localization of signaling molecules to primary cilia is important for activating downstream signaling pathways^22,23,57^. Therefore, the changes in the length of primary cilia are thought to be reflective of their functional modification. Indeed, the length of osteocyte primary cilia has been shown to correlate with their sensitivity to the mechanical stimuli^58^. In addition, several known human ciliopathies present with primary cilia of abnormal lengths^59^. In mouse femoral chondrocytes, primary cilia shorten in response to changes in extracellular osmolarity^40^. Likewise, in kidney epithelial cells as well as bone mesenchymal cells, fluid sheer-mediated bending of primary cilia results in decreased intracellular cAMP, which in turn causes shortening of primary cilia^41^. It is, therefore, possible that the changes in the length of primary cilia in NP cells under different osmotic conditions indicate altered intracellular signaling pathways. However, the pathway mediating the length of cilia in response to osmotic stimuli may be completely independent of the TonEBP-mediated osmotic response.

Our *Ift88* and *Kif3a* knockdown studies showed that the expression of TonEBP and its target genes was unaffected by the absence of primary cilia in NP cells. Under hyperosmotic conditions, TonEBP inhibits excessive sodium influx by increasing the levels of intracellular non-ionic osmolytes including taurine, sodium/myo-inositol, and betaine by inducing transcription of *TauT/Slc6a6*, *Bgt1*, *SMIT/Slc5a3*, and *AR/Akr1b1*^15–18^. Therefore, disruption of TonEBP activity under hyperosmotic condition can compromise cell survival. The fact that both *Ift88* and *Kif3a* knockdown did not affect the hyperosmotic induction of TonEBP and its target genes suggests that this was the result of the absence of primary cilia rather than a specific effect of the knockdown of either gene. Interestingly, despite the decrease in TonEBP protein levels, the expression of its transcriptional targets, *TauT/Slc6a6*, *SMIT/Slc5a3*, and *AR/Akr1b1* was not significantly affected under hypoosmotic conditions. This may be due to the fact that the osmoregulatory role of TonEBP is more important under hyperosmotic rather than hypoosmotic condition. Nonetheless, neither *Ift88* nor *Kif3a* silencing significantly affected how these genes were regulated in response to hypoosmotic stimuli, strongly suggesting the lack of primary cilia’s involvement in this process. In addition, the proximal promoter activities of select TonEBP targets, as well as TonEBP-TAD activity, were mostly unaffected by Sh*Ift88* or Sh*Kif3a* regardless of the osmotic status, suggesting that primary cilia play a minimal role, if any, in osmotic response of NP cells.

Our parallel studies using MEFs further confirmed that primary cilia do not control TonEBP-dependent osmotic response. The use of *Kif3a* null MEFs was advantageous mainly because they completely lack primary cilia, and because MEFs have TonEBP-dependent osmoregulatory pathways similar to that of NP cells^8,54^. Interestingly, unlike NP cells, *Kif3a* null MEFs were unable to upregulate *TauT* mRNA in response to the hyperosmotic stimulus. This was intriguing since *TauT* promoter activity was not affected by the absence of primary cilia in *Kif3a* null MEFs. This is perhaps due to the high sensitivity of luciferase reporter assay and the fact that it measures the activities of shorter promoter fragments. We cannot exclude the possibility of involvement of either a regulatory sequence outside the analyzed promoter fragment or a primary cilia-associated post-transcriptional regulation. Nonetheless, other osmoresponsive TonEBP targets, *SMIT/Slc5a3* and *AR/Akr1b1*, were upregulated under the hyperosmotic condition in *Kif3a* null MEFs, indicating that the overall cellular osmotic response to hyperosmotic stimuli is intact. Furthermore, we did not observe any obvious increase in *Kif3a* null MEF cell death in response to hyper- or hypo-osmotic stimuli, indicating that these cells were able to maintain their cellular homeostasis without primary cilia. The results of our *Kif3a* null MEFs are particularly interesting because the importance of primary cilia in modulating cell signaling during embryonic development is well-known.

Our data clearly demonstrate that TonEBP-mediated osmoregulation is not controlled by primary cilia in both NP cells and MEFs. The previously reported osmoregulatory and osmosensing function of primary cilia in cholangiocytes^36^ and kidney epithelial cells^38^ are therefore likely cell-type specific. Although the *in vitro* culture system is a simplification of the complex *in vivo* extracellular environment, the osmoregulatory role of primary cilia in other cell types was shown to be preserved in *in vitro* settings, suggesting that primary cilia are indispensable in these cell types. On the other hand, the results of our studies indicate that NP cells are capable of initiating TonEBP-mediated cellular osmoresponse without primary cilia. A recent study showed that mouse NP tissue cultured in an *ex vivo* setting decreased their TRPV4 expression in response to cyclic hyperosmolarity^60^. Future studies examining the localization and function of ion channels such as TRPV4, TRM3, or PC-1 and PC-2 that have been associated with cilia osmosensing would be helpful in better understanding how NP cells sense osmotic stimuli.

## Materials and Methods

### Reagents and plasmids

Lentiviral Sh*Ift88* #1 (TRCN0000178064), Sh*Ift88* #2 (TRCN0000182620), Sh*Kif3a* #1 (TRCN0000339512), Sh*Kif3a* #2 (TRCN0000339514), and control shRNA plasmids were purchased from Sigma. psPAX2 (12260) and pMD2.G (12259) developed by Dr. Didier Trono were obtained from Addgene. Luciferase reporter plasmids were kindly provided by Dr. Takashi Ito, Osaka University (wild type and TonE-mutant TauT-Luc)^15^, Dr. H Moo Kwon, University of Maryland (wild type and TonE-mutant HSP70-Luc)^20^, and Dr. Joan D. Ferraris, NIH (GAL4dbd-548-1531, GAL4dbd, and AR-Luc)^47,48^. Backbone GAL4dbd contains no TAD but only expresses the GAL4dbd. pFR-Luc reporter (Stratagene) contains the yeast GAL4-binding site, upstream of a minimal promoter driving the firefly luciferase gene.

### Cell culture and treatments

All procedures regarding collection of animal tissues was performed as per approved protocols by Institutional Animal Care and Use Committee (IACUC) of the Thomas Jefferson University, in accordance with the IACUC’s relevant guidelines and regulations. Rat NP cells were isolated using a method described by Risbud *et al*.^61^. After isolation, cells were maintained in Dulbecco’s Modified Eagles Medium (DMEM) (Corning, 10-013-CV) with 10% fetal bovine serum (FBS) (Sigma-Aldrich, F6178) supplemented with antibiotics in flask in normoxia (20.9% pO_2_). NP cells isolated from two rats were pulled into one T25 flask (P0) and cultured till confluency before passaging (P1). From this point, cells were either plated directly into experiment-specific plates (P2) or passaged. Approximately total of 16 rats were used for the study. Cells from different isolations were used for replication experiments. Cells up to P4 were used for the experiments. Wild type and *Kif3a*^−/−^ MEFs^54^ developed by Dr. Pao-Tien Chuang were kindly provided by Dr. Natalia Riobo, Thomas Jefferson University. For hyper- or hypoosmotic treatment, cells were cultured in DMEM with 10% FBS, containing either no additional osmolytes (330 mOsm/kg H_2_O), additional NaCl (60-135 mM to final osmolarity ranging 450–600 mOsm/kg H_2_O) or additional dH_2_O (to final osmolarity of 200 mOsm/kg H_2_O) for 4–24 h. For the hyperosmolarity experiments using NP cells, osmolarity ranging 450 to 550 mOsm/kg H_2_O have been used. While the estimated osmolarity of NP tissue *in vivo* ranges from 430 to 496 mOsm/kg H_2_O^3,4^, the above osmolarity range was chosen based on the previous reports on hyperosmotic TonEBP activation in NP cells^8,11,62^. Similarly, 600 mOsm/kg H_2_O was used for MEFs experiments based on the previous report on TonEBP/NFAT5 activity in MEFs, where the peak TonEBP-responsive promoter activity was observed at ~610 mOsm/kg H_2_O^63^.

### Real-time RT-PCR analysis

Total RNA was extracted from NP cells using RNAeasy mini columns (Qiagen). The purified, DNA-free RNA was converted to cDNA using RNA to cDNA EcoDry^TM^ Premix (Clontech). Template cDNA and gene specific primers were added to SYBR green master mix (Applied Biosystems) and mRNA expression was quantified using the Step One Plus Real-Time PCR System (Applied Biosystems). *Hprt1* was used to normalize gene expression. All the primers used were synthesized by Integrated DNA Technologies, Inc. (Coralville, IA).

### Transfections and dual luciferase assay

Cells were plated on 48-well plates (2 × 10^4^ cells/well) one day before transfection. Cells were transfected with 250 ng of wild type TauT, mutant TauT, wild type HSP70, mutant HSP70, or AR reporter plasmid and 250 ng of pRL-TK plasmid. For measuring TonEBP-TAD activity, cells were transfected with 200 ng of pFR-Luc, 200 ng of GAL4dbd-548-1531 (Ton-TAD) and 100 ng of pRL-TK plasmids. For each transfection, plasmids were premixed with the transfection reagent Lipofectamine 2000 (Invitrogen). The treatments were done so that the cells were lysed 48 h after the transfection. Dual-Luciferase^TM^ reporter assay system (Promega) was used for sequential measurements of firefly and Renilla luciferase activities using TECAN Infinite200 Pro microplate reader (TECAN). At least three independent transfections were performed and all analyses were carried out in triplicate.

### Immunofluorescence microscopy

NP cells were plated on poly-L-lysine-coated glass coverslips. After treatments, cells were fixed and permeabilized with 4% paraformaldehyde at room temperature for 15 minutes, washed with PBS and then blocked with 5% normal goat serum in PBS with 0.3% Triton X-100 (Sigma Aldrich, T8787) for 1 h at room temperature. Cells on coverslip were then incubated with anti-acetyl-α-tubulin (Lys40) (D20G3) XP® antibody (Cell Signaling Technology, #5335, 1:700), or anti-acetylated-α-tubulin (Sigma, T6793, 1:300) and anti-γ-tubulin antibody (Abcam, ab11317, 1:1000) for co-staining in blocking buffer at 4 °C overnight, washed with PBS, and then incubated with Alexa Fluor®-594 conjugated anti-rabbit, or Alexa Fluor®-594 conjugated anti-mouse, or Alexa Fluor®-488 conjugated anti-rabbit secondary antibody (Jackson ImmunoResearch Lab, Inc.), at a dilution of 1:700 for 1 h at room temperature in dark. Then the coverslips were washed with PBS and mounted with ProLong® Gold Antifade Mountant with DAPI (Thermo Fisher Scientific, P36934). Mounted slides were visualized using a Zeiss AxioImager A2 (Carl Zeiss, Germany), or Zeiss LSM510 confocal microscope (Carl Zeiss, Germany). Three independent experiments were performed for quantitative analysis of cilia length and incidence using ImageJ software (http://rsb.info.nih.gov/ij/). Briefly, each cilium was traced using Segmented Lines of the Line Selection Tools, and then the length of the tracing was measured using Measure function. The lengths of at least 150 primary cilia per treatment group were measured.

### Protein extraction and western blotting

Following treatment, cells were immediately placed on ice and washed with ice-cold PBS. All the wash buffers and the final cell lysis/re-suspension buffers included 1X complete^TM^ Mini Protease Inhibitor Cocktail (Roche, 11836153001), NaF (5 mM) (Sigma Aldrich, 201154) and Na_3_VO_4_ (200 μM) (Sigma Aldrich, S6508). Total cell proteins were resolved by electrophoresis on 8–12% SDS-polyacrylamide gels and transferred by electroblotting to PVDF membranes (EMD Millipore, IPVH00010). Dual-color Lane marker (Bio-Rad, 161-0394) was used as a protein ladder to guide identification of the band sizes. The membranes were blocked with 5% non-fat dry milk in TBST (1% Tween 20 in TBS) and incubated overnight at 4 °C in 5% non-fat dry milk in TBST with the antibodies against IFT88 (Proteintech, #13967-1-AP, 1:500), KIF3A (D7G3) (Cell Signaling Technology, #8507, 1:1000), TonEBP/NFAT5 (Novus Biologicals, NB120-3446, 1:1000), or GAPDH (Novus Biologicals, NB300-221, 1:3000). Immunolabeling was detected using the Amersham^TM^ ECL^TM^ Prime Western Blotting Detection Reagent (Thermo Fisher Scientific, 45-002-401). All Western blot experiments were performed at least three independent times.

### Lentiviral particle production and viral transduction

HEK 293 T cells (ATCC, CRL-3216) were plated in 10 cm plates (5 × 10^6^ cells/plate) in DMEM with 10% heat-inactivated FBS one day before transfection. Cells were transfected with 9 μg of Sh*Ctr*, Sh*Ift88*, or Sh*Kif3a* plasmids along with 6 μg psPAX2 and 3 μg pMD2.G using Lipofectamine 2000 (Invitrogen). After 6 h, transfection medium was replaced with DMEM with 10% heat-inactivated FBS and penicillin-streptomycin. Lentiviral medium was harvested at 48 to 60 h post-transfection, and mixed with 7% PEG 6000 (Sigma Aldrich, 81253) solution and incubated overnight at 4 °C to precipitate virus particles. PEG solution was removed from virus medium before transduction by centrifugation at 1,500 × *g* for 30 min to pellet virus particles. NP cells were plated in DMEM with 10% heat-inactivated FBS one day before transduction. Cells were transduced with fresh DMEM with 10% heat-inactivated FBS containing viral particles along with 8 μg/ml polybrene (Sigma Aldrich, H9268). After 16 h, the medium was removed and replaced with DMEM with 10% FBS. Cells were harvested for protein extraction 4–5 days after transduction to ensure maximum knockdown efficiency without affecting cell viability. At least three independent experiments were performed.

### Statistical analysis

All experiments were performed at least three independent times. Data are presented as the mean ± SE. Differences between multiple groups were assessed by one-way ANOVA or Kruskal-Wallis test depending on the distribution of the data with Sidak’s, Holm-Sidak’s, or Dunn’s multiple comparison test for post-hoc analyses using Prism7 (GraphPad Software). Some data were log-transformed before statistical analyses to account for non-Gaussian distribution. *P* < 0.05 was considered statistically significant.

## Supplementary information

Supplementary Info
