# Supplementary material for: Nucleus pulposus primary cilia alter their length in response to changes in extracellular osmolarity but do not control TonEBP-mediated osmoregulation
Source: Sci Rep. 2019 Oct 29;9:15469. doi: 10.1038/s41598-019-51939-7 (PMC6820757; doi:10.1038/s41598-019-51939-7)

Supplementary Information

Nucleus pulposus primary cilia alter their length in response to changes in extracellular osmolarity but do not control TonEBP-mediated osmoregulation

Hyowon Choi[1,2], Vedavathi Madhu[1], Irving M. Shapiro[1,2], Makarand V. Risbud[1,2*]

[1]Department of Orthopaedic Surgery, Sidney Kimmel Medical College, Thomas Jefferson University, Philadelphia, PA, USA
[2]Graduate Program in Cell Biology and Regenerative Medicine, Thomas Jefferson University, Philadelphia, PA, USA

Supplementary Figure S1-1. Uncropped images of Western blots.
Uncropped western blot images for Figure 2b, 2e, 3b, and 3e.

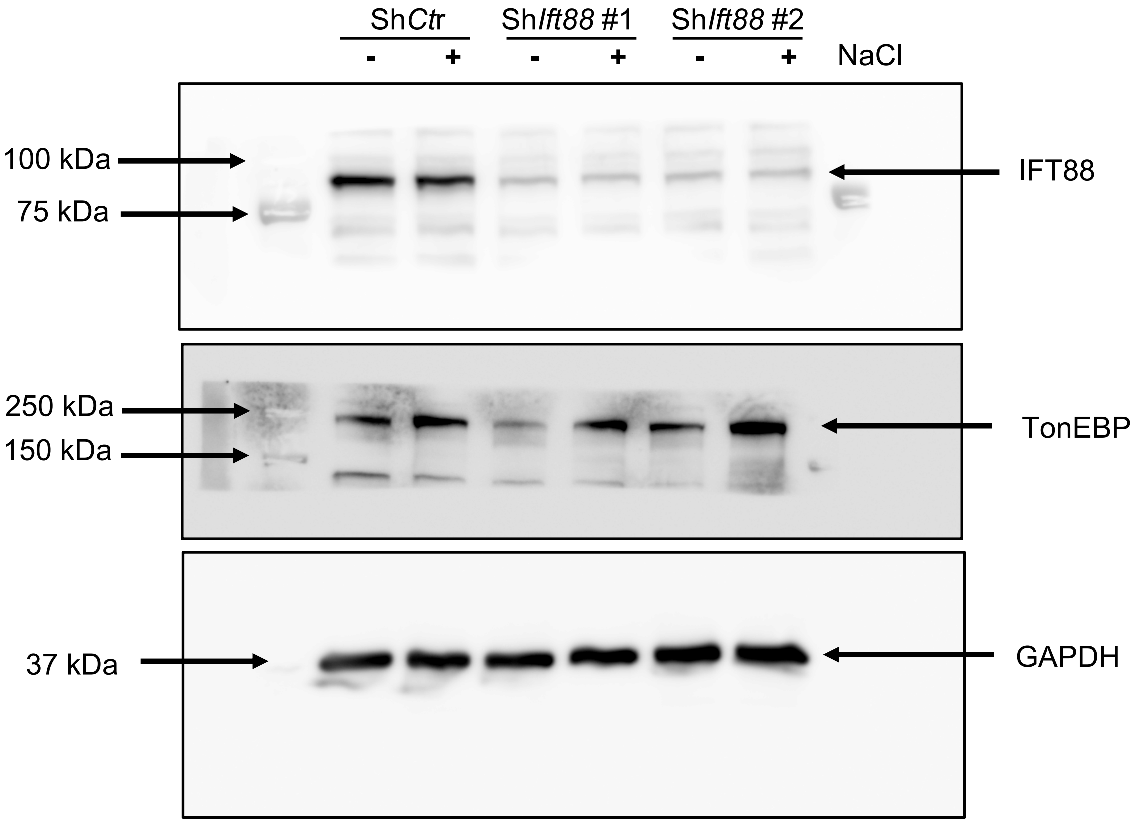

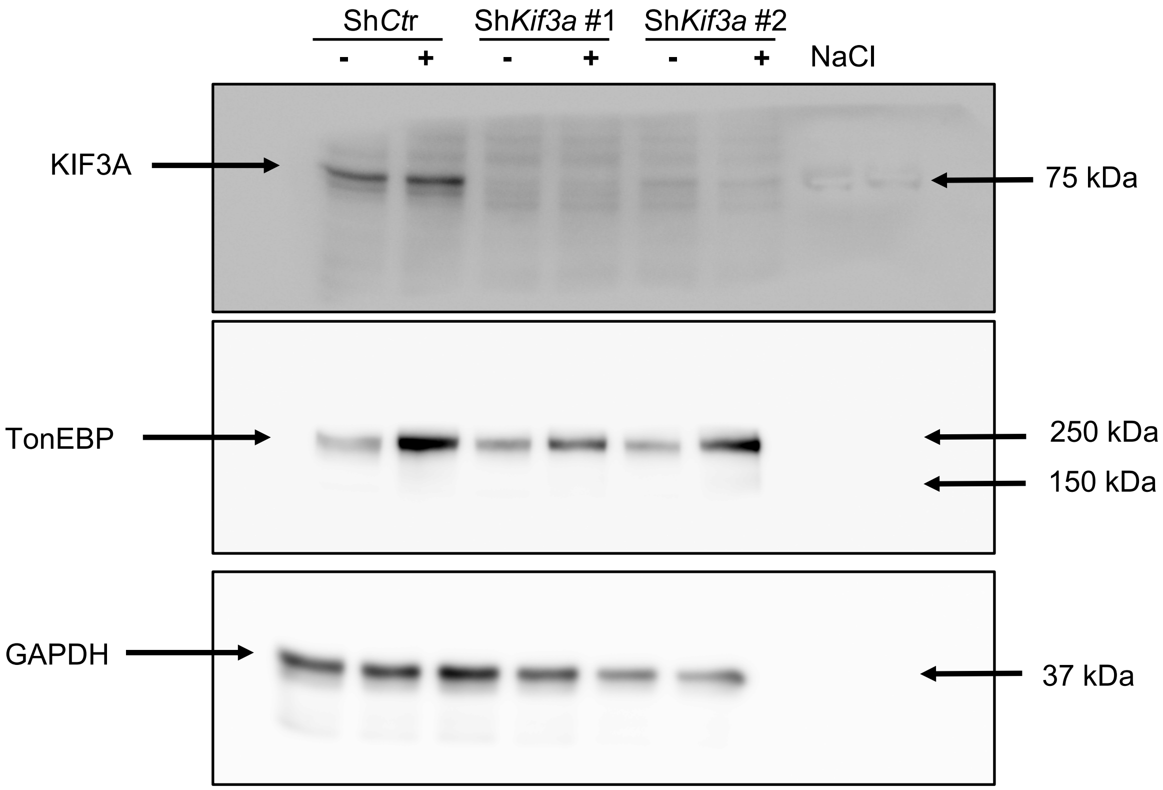

Supplementary Figure S1-2. Uncropped images of Western blots.
Uncropped western blot images for Figure 6b and 6g.

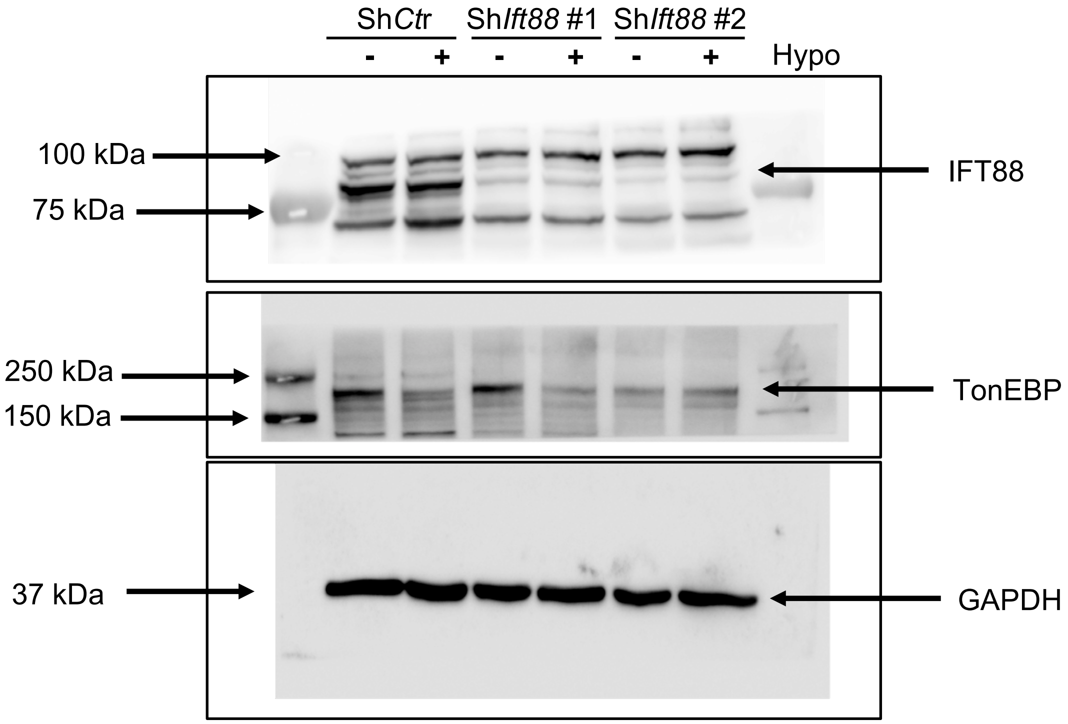

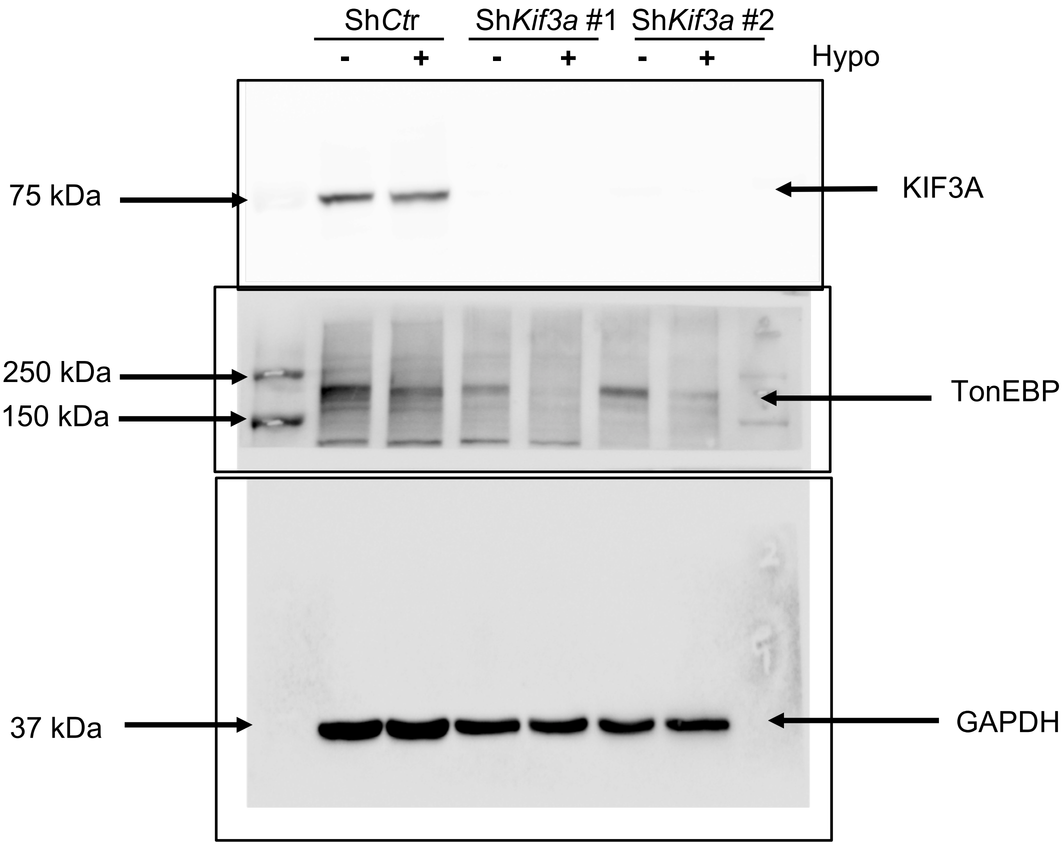

Supplementary Figure S1-3. Uncropped images of Western blots.
Uncropped western blot images for Figure 8b.

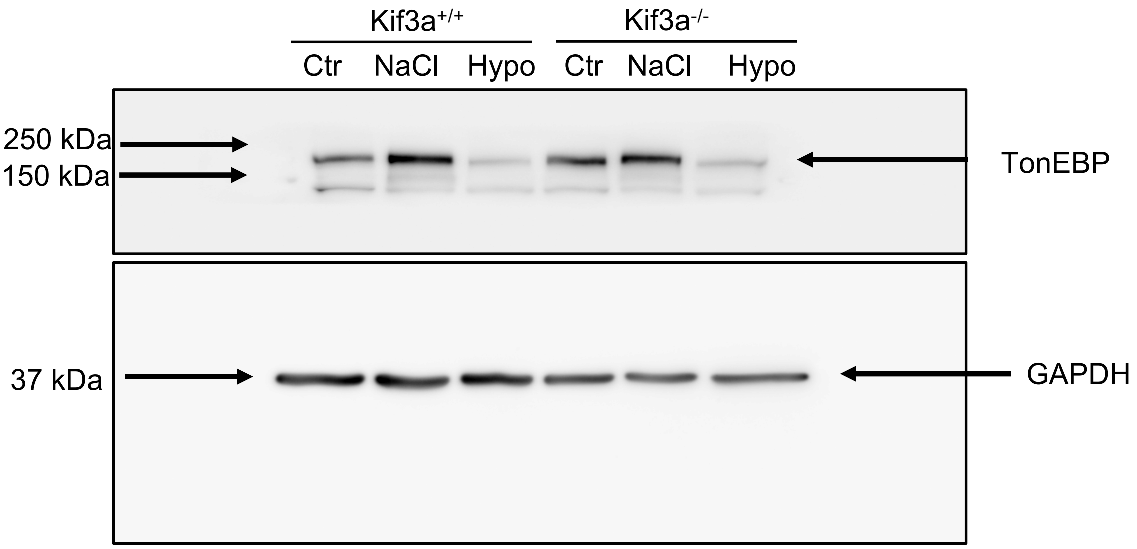

Supplement: Supplementary file 1 — Supplementary Info [file 41598_2019_51939_MOESM1_ESM.pdf]
